# Tropoelastin-Pretreated Exosomes from Adipose-Derived Stem Cells Improve the Synthesis of Cartilage Matrix and Alleviate Osteoarthritis

**DOI:** 10.3390/jfb14040203

**Published:** 2023-04-06

**Authors:** Shuo Meng, Cong Tang, Muhai Deng, Jie Yuan, Yanli Fan, Shasha Gao, Yong Feng, Junjun Yang, Cheng Chen

**Affiliations:** 1 College of Medical Informatics, Chongqing Medical University, Chongqing 400016, China; 2 Department of Orthopaedic Surgery, Chongqing Emergency Medical Center, Chongqing University Central Hospital, Chongqing 400014, China; 3 Key Laboratory of Biorheological Science and Technology, College of Bioengineering, Chongqing University, Ministry of Education, Chongqing 400044, China

**Keywords:** tropoelastin, adipose-derived stem cells, exosomes, articular cartilage, osteoarthritis

## Abstract

Mesenchymal stem cells (MSCs) have recently been widely used to treat osteoarthritis (OA). Our prior research shows that tropoelastin (TE) increases MSC activity and protects knee cartilage from OA-related degradation. The underlying mechanism might be that TE regulates the paracrine of MSCs. Exosomes (Exos), the paracrine secretion of MSCs, have been found to protect chondrocytes, reduce inflammation, and preserve the cartilage matrix. In this study, we used Exos derived from TE-pretreated adipose-derived stem cells (ADSCs) (TE-Exo^ADSCs^) as an injection medium, and compared it with Exos derived from unpretreated ADSCs (Exo^ADSCs^). We found that TE-Exo^ADSCs^ could effectively enhance the matrix synthesis of chondrocytes in vitro. Moreover, TE pretreatment increased the ability of ADSCs to secrete Exos. In addition, compared with Exo^ADSCs^, TE-Exo^ADSCs^ exhibited therapeutic effects in the anterior cruciate ligament transection (ACLT)-induced OA model. Further, we observed that TE altered the microRNA expression in Exo^ADSCs^ and identified one differentially upregulated microRNA: miR-451-5p. In conclusion, TE-Exo^ADSCs^ helped maintain the chondrocyte phenotype in vitro, and promoted cartilage repair in vivo. These therapeutic effects might be related with the altered expression of miR-451-5p in the Exo^ADSCs^. Thus, the intra-articular delivery of Exos derived from ADSCs with TE pretreatment could be a new approach to treat OA.

## 1. Introduction

Osteoarthritis (OA) is a common degenerative joint disease that leads to pain, limited mobility, and disability, with total joint arthroplasty as the ultimate solution [1]. Extracellular matrix (ECM) depletion and articular cartilage degradation are characteristics of OA [2]. Many risk factors, including genetics, gender, trauma, age, and obesity, are implicated in OA. Approximately 300 million people suffer from OA globally [3], and the primary pathologic alteration of OA is articular cartilage lesion [4]. Hence, the primary strategy for preventing the development of OA is to promote articular cartilage repair or regeneration. It remains complicated to regenerate or repair cartilage using current therapeutic techniques such as medications [5], physical therapy [6], microfracture [7], or cartilage transplantation [8].

Tissue engineering and regenerative medicine have advanced significantly over the last several years [9], and these developments provide promising approaches to contribute to articular cartilage regeneration. Many studies have demonstrated that defects in articular cartilage may be efficiently restored in vitro or in vivo, including mesenchymal stem cells (MSCs) [10] and other tissue engineering techniques [11]. Both primary and clinical research has shown that MSC-based therapies are reliable and effective for cartilage regeneration [12,13,14]. MSCs isolated from bone marrow, adipose tissue, umbilical cord, or amniotic fluid could all be used for cartilage repair and OA treatment [14,15]. In particular, adipose-derived stem cells (ADSCs) have become a potential and promising source of MSCs [16]. Even so, therapeutic uses of MSCs have technical limitations regarding cell sources, donor site morbidity, the time required for cell expansion, low retention, and the risk of tumorigenicity or infection [17].

Recently, the underlying mechanism of MSC-based therapeutic approaches for treating OA gradually changed from the ability of MSCs to engraft and differentiate to the impact of the paracrine mechanism [17]. The pro-regenerative properties of MSC secretomes have been reported in various systems, including the inhibition of cell death and fibrosis, modulation of the immune system, stimulation of angiogenesis, promotion of tissue regeneration, and homing cells [18]. As a result, recently developed alternative techniques have used non-cellular products including exosomes (Exos) [19,20], extracellular vesicles (EVs) [21], micro-RNA (miRNA) [22], and platelet lysate [23] to substitute traditional cell-based OA therapies. Exos, which are small (30–150 nm in diameter) vesicles secreted by cells, are known as an intermediate mediator of various paracrine effects. Exos can mediate communications between cells and modulate various biological processes, including immune response and inflammation [24]. Exos include a variety of biological components such as proteins, lipids, mRNA, and miRNA that are comparable to those found in the original parent cells [25]. Exos released by various cell types, and even by the same cell type under various circumstances, vary significantly in their components. Exos isolated from cells have been shown in several studies to have a more focused impact when pretreated using particular techniques [26]. Tropoelastin (TE), a 60–72 kDa alternatively spliced ECM protein, is prevalent in all vertebrate species except cyclostomes [27]. As a soluble additive, TE could control MSCs’ adherence, proliferation, and phenotypic maintenance [28]. Our previous study demonstrated that TE inhibits the development of OA and enhances the adhesion and migration of infrapatellar fat pad MSCs injected intra-articularly [29]. Therefore, it is essential to investigate the paracrine alterations caused by the TE pretreatment of MSCs. In this study, we pretreated rat ADSCs with TE and harvested their Exos to compare with Exos derived from unpretreated ADSCs (Exo^ADSCs^). The main objective is to investigate whether Exos from TE-pretreated ADSCs (TE- Exo^ADSCs^) have better outcomes in promoting cartilage ECM both in vitro and in vivo and to identify the possible mechanisms involved.

## 2. Materials and Methods

### 2.1. Isolation and Culture of Primary Chondrocytes

Following the previously reported approach, primary articular chondrocytes (ACs) were separated from knee articular cartilage fragments harvested from the femoral condyles and tibial plateaus of 8-week-old Sprague Dawley (SD) female rats [30]. Briefly, the cartilage pieces were minced and digested with 0.2% type II collagenase (Biosharp, Guangzhou, China) overnight at 37 °C. The cell suspension was then passed through a 40 µm cell strainer (Millipore, Burlington, MA, USA). After being centrifuged (200× *g* for 5 min), the ACs were resuspended in DMEM/high glucose medium (Gibco, New York, NY, USA) containing 10% FBS (Gibco, New York, NY, USA) and 1% penicillin–streptomycin (P/S) (Beyotime, Shanghai, China). Upon confluency, the ACs were further sub-cultured to passage 2 for the following experiments.

### 2.2. ADSC Culture and Pretreatments

Rat ADSCs were purchased from Cyagen Biosciences (Guangzhou, China) Inc. (cat. RASMD-01001). The ADSCs were cultured in DMEM/F12 (Gibco) supplemented with 10% FBS and 1% P/S. The cells were maintained and expanded by 3–5 passages before use. To obtain Exos, the ADSCs were cultured using DMEM/F12 supplemented with 10% exosome-depleted FBS (VivaCell, Shanghai, China). The ADSCs were unpretreated or pretreated with tropoelastin (TE) for 48 h to prepare Exo^ADSCs^ or TE-Exo^ADSCs^. The conditioned medium of ADSCs was harvested and centrifuged at 3000× *g* for 10 min to remove cells and debris.

### 2.3. Preparation and Characterization of Exo^ADSCs^ and TE-Exo^ADSCs^

Exo^ADSCs^ and TE-Exo^ADSCs^ were isolated from the ADSC supernatant using total exosome isolation reagent (Thermo Fisher, Waltham, MA, USA) according to the manufacturer’s instructions. Briefly, 1 mL of conditioned medium was mixed with 500 µL total exosome isolation reagent and incubated overnight at 4 °C. Subsequently, the mixture was centrifuged at 10,000× *g* for 1 h at 4 °C, and the supernatant was aspirated and discarded. The exosome pellet was resuspended in PBS.

Transmission electron microscopy (TEM) was performed to observe exosome morphology. The size distribution and particle concentration of Exo^ADSCs^ and TE-Exo^ADSCs^ were analyzed using nanoparticle tracking analysis (NTA). Western blot analysis was used to analyze the exosomal surface markers, such as CD81 (1:1000, Abcam), TSG101 (1:1000, Abcam) and Calnexin (1:1000, Abcam). ADSCs were used as the negative control.

### 2.4. Distribution of Exo^ADSCs^ and TE-Exo^ADSCs^ in ACs

In order to examine the cellular uptake of Exos, unpretreated or pretreated ADSCs were labeled using DiO for 48 h at 37 °C, and then the labeled Exos were collected from the supernatant. The ACs were seeded into plates and incubated with DiO-labeled Exos for 48 h at 37 °C. Then, the ACs were fixed in 4% paraformaldehyde and sequentially stained with rhodamine–phalloidin and DAPI solution. A laser scanning confocal microscope (LSM780 ZEISS) was used to observe the distributions of Exo^ADSCs^ or TE-Exo^ADSCs^.

### 2.5. Potential of Exos on ECM Synthesis and Its Effect on Phenotype of ACs in Co-Culture

To further clarify the potential of TE-Exo^ADSCs^ on ECM synthesis, ACs (5 × 10^4^ cells) were seeded into cell slides filled with Exo^ADSCs^, TE-Exo^ADSCs^, TE+ Exo^ADSCs^, or TE solution (20 µg/mL). The cells were incubated for 48 h at 37 °C, and the expression of collagen II (COL Ⅱ), SOX 9, and aggrecan (ACAN) was measured with immunofluorescence staining and Western blot.

### 2.6. Immunofluorescence Staining

Next, the fixed cells were incubated overnight at 4 °C with primary antibodies (COL II, SOX 9, and ACAN) (1:1000, Abcam), followed by incubation with secondary antibodies (Ms-647 or Rb-488) (1:1000, Abcam) for 60 min at room temperature. The nuclei were stained with DAPI (Beyotime) for 10 min, and the samples were then observed using a laser scanning confocal microscope. The relative fluorescence unit (RFU) was analyzed using ZEN 2012 software, version 1.1.0.0 (Carl Zeiss Microscopy, Jena, Germany). For statistical analysis, we used three different fluorescent images from each group, calculating the total fluorescence intensity of the image divided by the number of nuclei.

### 2.7. Western Blot Analysis

To extract total protein from the ACs, we used RIPA lysis buffer (Beyotime, Shanghai, China) supplemented with protease–phosphatase inhibitors (Beyotime, Shanghai, China) on ice for 20 min, followed by centrifugation at 12,000× *g* for 30 min at 4 °C. We determined the protein concentration using the BCA protein assay kit (Beyotime, Shanghai, China) and separated 50 ng of protein using SDS-PAGE (Beyotime, Shanghai, China). The protein was subsequently transferred to a PVDF membrane (Millipore, Burlington, MA, USA). We blocked the membranes with QuickBlock blocking buffer overnight at 4 °C and then incubated them overnight at 4 °C with the primary antibodies (COL II, ACAN, SOX 9), followed by incubation with the respective secondary antibodies for 1 h at room temperature. After washing the membranes three times with TBST, we visualized them using the SuperSignal West Femto kit (Thermo Fisher, Waltham, MA, USA). Finally, we quantified the intensity of the blots using Image Lab 3.0 software (Bio-Rad, Hercules, CA, USA).

### 2.8. Role of Exos in IL-1β-Induced Chondrocyte Inflammation

Chondrocytes were treated with IL-1β (10 ng/mL) to evaluate the impact of Exos in attenuating the inflammatory response. They were co-cultured for 48 h with Exo^ADSCs^, TE-Exo^ADSCs^, TE+ Exo^ADSCs^, or TE solution. The expression of ADAMTS-5 and MMP-13 was measured using Western blot.

### 2.9. Effect of Exo^ADSCs^ and TE-Exo^ADSCs^ on Cartilage Matrix Deposition in ACs

Glycosaminoglycans (GAGs) are the essential components in the ECM of cartilage tissue, which also promotes cartilage regeneration. The levels of GAGs released from the cultured chondrocytes were determined by Alcian blue and safranin O staining. The cultured monolayer chondrocytes on glass slides were fixed in 4% paraformaldehyde for 10 min and stained with hematoxylin (10 min) and eosin (1 min). The morphological characteristics of the chondrocytes and GAGs were observed by phase-contrast microscopy.

### 2.10. Induction of Rat OA

All animal experiments were approved by the animal research committee regulations of Chongqing Medical University (No. 2022070). Male Sprague Dawley rats (approximately 12 weeks old) weighing 300–350 g, housed in a specific pathogen-free (SPF) animal laboratory with 12:12 h light/dark cycle, controlled temperature environment (23–25 °C) and steady humidity (55–70%), were used in this study. The OA model was established by transecting the anterior cruciate ligament (ACLT). Four weeks after OA induction, the rats received an intra-articular injection of the Exos suspension.

### 2.11. Intra-Articular Injection of Rat Knee

To study the effects of Exos intra-articular injection therapy, we randomly assigned six groups of SD rats as follows: (1) normal group (PBS injection; *n* = 5); (2) OA group (PBS injection, *n* = 5); (3) OA + Exo^ADSCs^ group (Exo^ADSCs^ injection, *n* = 5); (4) OA + TE-Exo^ADSCs^ group (TE-Exo^ADSCs^ injection, *n* = 5); (5) OA + TE + Exo^ADSCs^ group (Exo^ADSCs^ injection, *n* = 5); and (6) OA + TE group (TE injection, *n* = 5). After eight weeks post-surgery, we sacrificed the rats by anesthetic overdose and collected knee samples to assess the progression of OA.

### 2.12. Histologic Evaluation

We followed a specific protocol for the histological analysis of the rat knee joints. First, we sacrificed the rats and fixed their knee joints in 4% paraformaldehyde. Then, we decalcified the specimens in 10% EDTA and embedded them in paraffin. To ensure the best serial sagittal sections, including the whole joint, we collected 5 μm sections at 50 μm intervals. The paraffin sections were subjected to HE staining, safranin O-fast green staining, and immunohistochemical staining of COL II. We evaluated the histological changes in the rat knee joints using the Osteoarthritis Research Society International (OARSI) scoring system [31], Mankin scoring systems [32], and synovitis scores [33]. Lastly, we examined the relative staining intensity of COL II and ACAN in three core locations of the articular cartilage using Fiji version 2.9 software.

### 2.13. Exosomal miRNA Sequencing

We performed miRNA sequencing on both Exo^ADSCs^ and TE-Exo^ADSCs^. To identify differentially expressed miRNAs, we set a fold change threshold of >1 and *p*-value threshold of <0.05 for the up- and downregulated genes.

### 2.14. Statistical Analysis

We conducted the statistical analysis using GraphPad Prism software, version 9.0 (GraphPad Software, San Diego, CA, USA). For multiple group comparisons with parametric data, we performed a one- or two-way ANOVA with Tukey’s post hoc test. We considered a *p*-value < 0.05 to be statistically significant. All data are presented as mean ± standard error of the mean (SEM).

## 3. Results

### 3.1. Exo^ADSCs^ and TE-Exo^ADSCs^ Have Similar Characteristics

**Figure 1:**
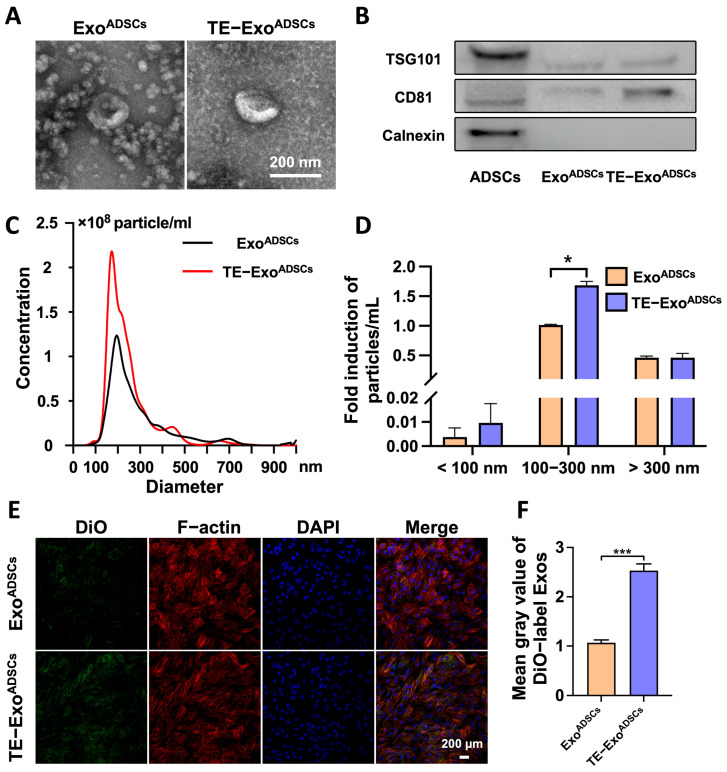
TE promoted Exos release from ADSCs. (**A**) Morphology of Exo^ADSCs^ and TE-Exo^ADSCs^ under TEM. (**B**) Western blot analysis of exosomal proteins including TSG101, CD81, and Calnexin. (**C**) NTA analysis of Exo^ADSCs^ and TE-Exo^ADSCs^ showed the particle concentrations and size ranges of Exos in the two groups. (**D**) The size distribution of Exo^ADSCs^ and TE-Exo^ADSCs^. (* *p* < 0.05). (**E**) Uptake of the green fluorescence dye DiO-labeled Exo^ADSCs^ and TE-Exo^ADSCs^ into chondrocyte. (**F**) Fluorescence intensities in the two groups were statistically evaluated (*n* = 5, *** *p* < 0.001).

To investigate the effect of TE pretreatment on the characteristics of Exos, we analyzed the yield, morphology, size distribution, and marker expression of Exo^ADSCs^ and TE-Exo^ADSCs^ (Figure 1). TEM analysis revealed that both groups contained typical Exos with homogeneous, spherical, and membrane-bound vesicles (Figure 1A). Western blot examination showed that these particles contained exosomal surface markers, including TSG101 and CD81, but were negative for the non-exosomal marker Calnexin (Figure 1B). The average value of particles per mL were 1.81 × 10^10^ and 2.68 × 10^10^ in Exo^ADSCs^ and TE-Exo^ADSCs^, respectively; the diameter of these Exos was 50–200 nm (Figure 1C), and the number of Exos with diameters between 0–100 nm and 100–300 nm was significantly increased (nearly 2.54-fold and 1.68-fold on average, respectively) in the TE-Exo^ADSCs^ compared to the Exo^ADSCs^, while the number of exosome-like vesicles with diameters in other ranges remained unchanged (Figure 1D). These results suggest that the TE pretreatment of ADSCs might lead to the increased production or release of 0–300 nm exosome-like vesicles. These results indicated that the Exos we isolated exhibited the characteristics of MSC-derived Exos and could be used for subsequent experiments.

Based on the NTA results, we diluted both types of exosomes to 1 × 10^10^ particles/mL with PBS. For the in vitro experiment, we treated each group of cells with culture medium containing 5 × 10^8^ particles/mL of exosomes. For the in vivo experiment, we injected 10 µL of Exos solution containing 1 × 10^10^ particles/mL into the joint cavity.

### 3.2. Exos Are Taken Up by Chondrocytes

To determine whether the chondrocytes directly took up the Exos, the Exos were labeled with the fluorescent dye DiO and then cocultured with chondrocytes for 48 h. We observed that the chondrocytes robustly took up more TE-Exo^ADSCs^ than Exo^ADSCs^ by assessing the amount of transferred fluorescence (Figure 1E,F). The results showed the presence of DiO-labeled Exos in the cytoplasm, confirming the uptake of Exos by the chondrocytes.

### 3.3. Exos Help Maintain Chondrocyte Phenotype In Vitro

**Figure 2:**
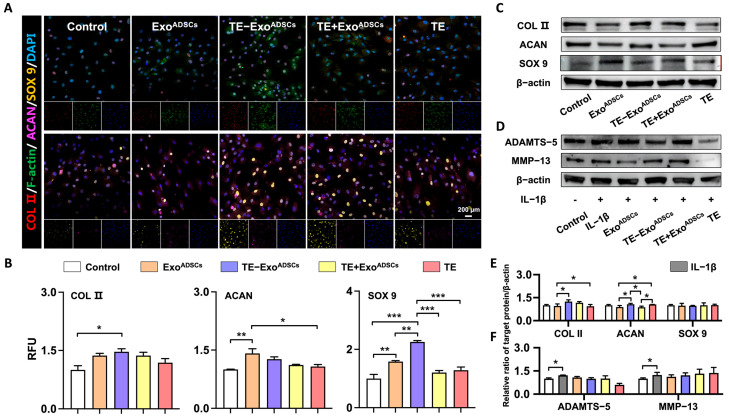
Exos maintained the phenotype chondrocytes in vitro. (**A**) Representative fluorescence images of COL II (red), F-actin (green), ACAN (purple), and SOX 9 (yellow). DAPI was used to stain the nuclei (blue). (**B**) Relative fluorescence unit (RFU) of COL II, ACAN, and SOX 9 was analyzed. (*n* = 5, * *p* < 0.05, ** *p* < 0.01, *** *p* < 0.001). (**C**,**E**) Representative Western blot of COL II, SOX-9, and ACAN. β-actin was used as a standard for loading in Western blots. (*n* = 3, * *p* < 0.05). (**D**,**F**) Representative Western blot of ADAMTS-5 and MMP-13. β-actin was used as a standard for loading in Western blots (*n* = 3, * *p* < 0.05).

To investigate the effect of Exos on cartilage-related proteins, we conducted in vitro experiments (Figure 2). The results showed that TE-Exo^ADSCs^ caused significant increases in COL II and SOX 9 compared to Exo^ADSCs^, as confirmed by immunofluorescence staining and RFU statistical analysis (Figure 2A,B). The ACAN and SOX 9 expressions were slightly higher in the Exo^ADSCs^ group compared to the control group. However, after treatment with TE-Exo^ADSCs^, the ACAN and COL II expression levels significantly increased compared to Exo^ADSCs^, according to Western blot analysis. There was no significant difference in SOX 9 expression among the five groups (Figure 2C,E).

We further studied the effectiveness of TE-Exo^ADSCs^ treatment on ACs in an IL-1β-induced in vitro model of OA. After IL-1β pretreatment, we measured the expression of ADAMTS-5 and MMP-13, as the two enzymes are mainly responsible for the breakdown of the cartilage matrix. Our results showed that the expression levels of MMP-13 and ADMATS-5 were significantly upregulated by IL-1β treatment, and this change was not reversed by the addition of either Exos or TE (Figure 2D,F). These results suggested that Exos treatment, especially TE-Exo^ADSCs^ and Exo^ADSCs^, might help maintain the chondrocyte phenotype, but cannot rescue the catabolic change induced by the proinflammatory cytokine IL-1β.

### 3.4. Exos Promote the Formation of Cartilage Extracellular Matrix

**Figure 3:**
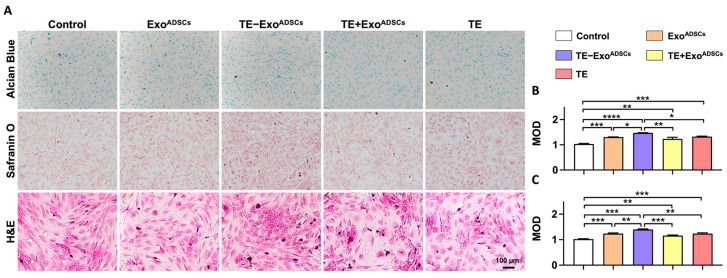
Exos promoted the formation of cartilage extracellular matrix. (**A**) Representative Alcian blue, safranin O, and H&E staining images of chondrocyte. DAPI was used to stain the nuclei. (**B**) Mean optical density (MOD) of Alcian blue-stained chondrocytes. (*n* = 5, * *p* < 0.05, ** *p* < 0.01, *** *p* < 0.001, **** *p* < 0.0001). (**C**) MOD of safranin O-stained chondrocytes (*n* = 5, ** *p* < 0.01, *** *p* < 0.001).

Alcian blue and safranin O staining were performed to verify the cartilage matrix deposition in the chondrocytes. As shown in Figure 3A–C, the TE-Exo^ADSCs^ group exhibited the strongest staining intensity and expressed increasing levels of GAGs compared to the control group. H&E staining revealed that the chondrocytes in the TE-Exo^ADSCs^ group displayed more noticeable blue nuclei that were ovoid, and pink cytoplasm that was triangular or spindle-shaped. These findings suggested that TE-Exo^ADSCs^ more effectively stimulated chondrocyte regeneration and cartilage ECM deposition.

### 3.5. TE-Exo^ADSCs^ Intra-Articularly Alleviate Rat OA

**Figure 4:**
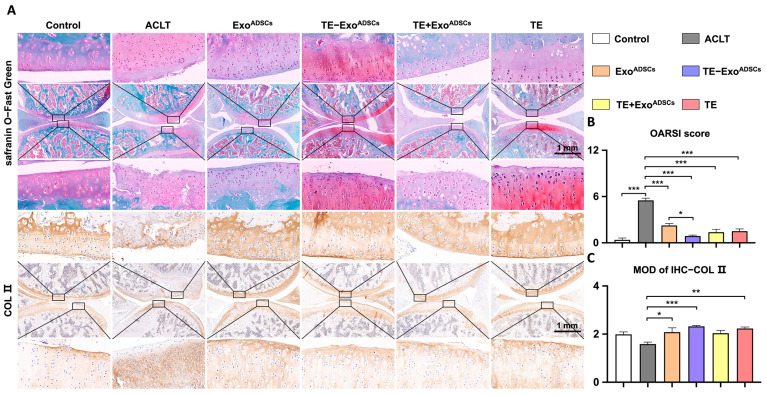
Intra-articular injection of TE-Exo^ADSCs^ effectively reduced rat OA. (**A**) Safranin O-Fast Green and COL II immunohistochemistry staining of rat knee joints. Images of femur and tibia were from black boxed area. In Safranin O-Fast Green staining images, red represents cartilage matrix, and green represents bone tissue. COL II positive areas in immunohistochemical staining images appear as brownish-yellow in color. (**B**) Statistical analysis of the OARSI score (safranin O-Fast Green staining) in rats (*n* = 5, * *p* < 0.05, *** *p* < 0.001). (**C**) Semi-quantitative results of immunohistochemical staining for COL II (*n* = 5, * *p* < 0.05, ** *p* < 0.01, *** *p* < 0.001).

**Figure 5:**
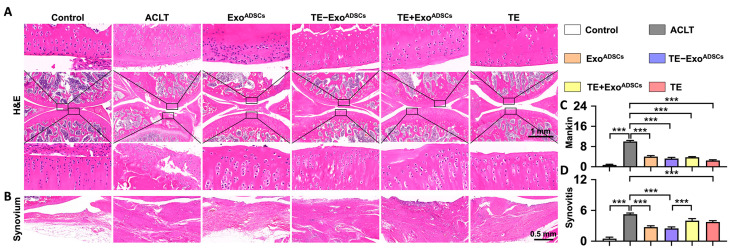
Intra-articular injection of TE-Exo^ADSCs^ effectively protected cartilage matrix and inhibited synovium inflammation. (**A**,**B**) H&E staining of rat knee joints and synovium. Images of femur and tibia were from black boxed area. In H&E staining images, red represents cytoplasm and blue represents cell nucleus. (**C**) Statistical analysis of the Mankin score in cartilages (*n* = 5, *** *p* < 0.001). (**D**) Statistical analysis of synovitis scores (*n* = 5, *** *p* < 0.001).

A rat model of OA induced by ACLT was utilized to examine the protective role of TE-Exo^ADSCs^ suspension on the articular cartilage. HE, safranin O/fast green, and COL II immunohistochemical stains were applied to identify cartilage structural damage and proteoglycan loss (Figure 4 and Figure 5). According to the example pictures in Figure 4A and Figure 5A, articular cartilage was significantly diminished following ACLT surgery. In contrast to the Exo^ADSCs^, TE+ Exo^ADSCs^, and TE injection groups, the TE-Exo^ADSCs^-treated knee joints of ACLT-induced OA rats exhibited full cartilage integration with a smooth surface, expression levels of ACAN, and the regular organization of chondrocytes.

The severity of cartilage damage was quantified using the OARSI scoring (Figure 4B). Exo^ADSCs^, TE-Exo^ADSCs^, TE+ Exo^ADSCs^, and TE restored the cartilage damage considerably following ACLT surgery. The Exo^ADSCs^, TE-Exo^ADSCs^, TE+Exo^ADSCs^, and TE group exhibited greater relative staining intensity than the ACLT group. In addition, an immunohistochemistry analysis was conducted to evaluate the variation of COL II in SD rat articular cartilage. Exo^ADSCs^, TE-Exo^ADSCs^, and TE restored the reduced expression of COL II in cartilage (Figure 4C).

The Mankin scoring system was used to assess cartilage damage (Figure 5C). The Mankin cartilage scores were enhanced after creating OA rat models. The Exo^ADSCs^, TE-Exo^ADSCs^, TE+ Exo^ADSCs^, and TE groups all had lower Mankin scores than the ACLT group.

The rat knee joints showed alterations in the synovial tissues (Figure 5B). Compared to the OA group, the rats in the TE-Exo^ADSCs^ group had less synovial tissue proliferation, inflammatory infiltration, and microvessels, albeit inflammatory infiltration was still present in large quantities. The intensity of inflammation in the synovium of the knee joints was graded (Figure 5D). The rats in the OA group showed high-grade inflammation, with ratings for synovitis that were worse than those in the other four groups. Our results indicated that TE-Exo^ADSCs^ decreased cartilage damage in the knee joints of OA rats.

### 3.6. TE Pretreatment Increased ADSC miR-451-5p Expression and Its Release via Exos

**Figure 6:**
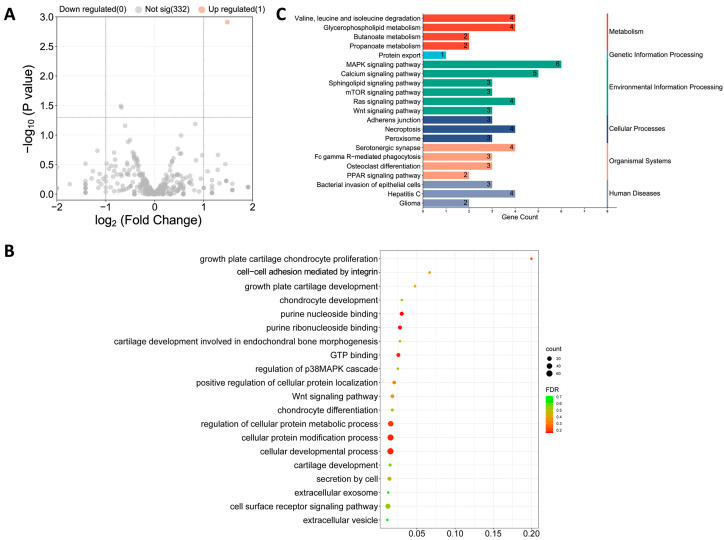
miR-451-5p was enriched in TE-Exo^ADSCs^, indicated by exosomal miRNA sequencing. (**A**) Volcano plot displaying the one significant differentially expressed miRNA based on the criteria of *p* < 0.05 and fold change > 1.0. (**B**) GO pathway enrichment analysis of the target genes of miR-451-5p. (**C**) KEGG pathway enrichment analysis of the target genes of miR-451-5p.

Accumulating evidence shows that miRNAs loaded in Exos are essential in executing their cellular function [34]. To examine the biological mechanism behind the enhanced rate of TE-Exo^ADSCs^ in cartilage protection, we performed miRNA sequencing on Exos secreted from ADSCs pretreated with or without TE to identify differentially expressed miRNA (Figure 6). As a result, miR-451-5p was identified to be upregulated (over 1.0-fold change) in TE-Exo^ADSCs^ compared to Exo^ADSCs^, and 232 potential target genes for miR-451-5p were identified (Figure 6A).

A total of 232 potential target genes for miR-451-5p were discovered using the miRanda database. The GO functions are shown in Figure 6B and are mainly associated with purine nucleoside binding; growth plate cartilage chondrocyte proliferation; secretion by cell; growth plate cartilage development; chondrocyte development; chondrocyte differentiation; extracellular exosome, and so on. The pathways of these target genes were investigated using KEGG pathway enrichment analysis (Figure 6C), which included adherens junction; MAPK signaling pathway; protein export; bacterial invasion of epithelial cells; valine, leucine, and isoleucine degradation; and serotonergic synapse. However, these biological functions and pathways in relation to OA progression and cartilage repair are less identified.

## 4. Discussion

Damage to articular cartilage is a significant pathogenic alteration in OA. We pretreated ADSCs with TE in this study and subsequently isolated Exos from the supernatant. Our findings suggested that TE-Exo^ADSCs^ may stimulate cartilage regeneration by altering the expression of miRNAs such as miR-451-5p.

MSCs have been used in several clinical trials to treat OA, and these studies have yielded excellent therapeutic results [35]. However, specific issues, such as technical limitations, still make collecting and storing the cells challenging. MSCs also decline in number and function as the donor ages [36]. MSCs have also been demonstrated to be temporary following systemic injection. Although MSCs have some capacity for differentiation, research has demonstrated that their paracrine activity, notably the secretion of Exos, is crucial for tissue healing [37]. Exos derived from MSCs are now used in several preclinical trials to treat various illnesses, including OA [38]. Exos derived from bone marrow stem cells have been demonstrated to efficiently stimulate cartilage cell proliferation, migration, and ECM formation in animal OA models, resulting in cartilage regeneration and decreased knee inflammation [39]. Nonetheless, the original Exos have a lesser targeting ability and a quicker drug clearance rate in vivo, which results in subpar therapeutic effectiveness [40]. Consequently, with modification or pretreatment, Exos are attractive delivery systems for targeted pharmaceuticals due to their intrinsic benefits.

As a soluble factor, TE could modulate cell behavior and remarkably protect against phenotypic variation within the MSC population [28]. We previously observed that TE stimulates IPFP-MSCs and protects knee cartilage from OA injury by promoting cellular adhesion, which may be associated with the paracrine function of MSCs [29]. The significance of TE-pretreated MSC-Exos in OA remains unknown. In order to investigate whether ADSCs-derived Exos could also benefit from TE treatment, we designed TE-Exos^ADSCs^ and TE+Exos^ADSCs^ treatment groups. The former group aimed to explore whether TE would induce differences in the secretion of Exos from MSCs, while the latter was to differentiate whether TE directly regulated the functional effects of Exos.

Here, the concentration of Exos was adopted from our previous study [34], in which we used 5 × 10^8^ particles/mL for the in vitro experiment and 10 µL of Exos solution containing 1 × 10^10^ particles/mL for the intra-articular injection. It has been shown that the higher dose of MSC-derived Exos promotes chondrocyte proliferation [41]. Therefore, we speculated that the dose of TE-Exo^ADSCs^ could possibly be optimized to obtain better results in terms of cartilage repair in future studies. Exos derived from the TE pretreatment and unpretreated groups did not exhibit any morphological variations in terms of size, shape, or electron density, according to the TEM and NTA data. However, this study showed that TE pretreatment increased the ability of ADSCs to release Exos. Exos stimulated ECM deposition in chondrocytes in vitro, while TE pretreatment enhanced these therapeutic advantages. The TE-pretreated group also had a superior therapeutic effect in vivo compared to the other groups. Note that, for the COL II immunohistochemistry staining results of the rat knee joints, all groups except for TE+Exo^ADSCs^ group showed significant differences with the ACLT group. This might be because the precursor TE becomes elastin in vivo, and the presence of elastin and its elastic fibers temporarily inhibits the expression of COL II [42].

We concentrated on distinguishing between Exos derived from TE-pretreated or TE-unpretreated ADSCs when we noticed the positive benefits of TE-Exo^ADSCs^ compared to Exo^ADSCs^. MicroRNAs are short, naturally occurring, non-coding RNAs that play a role in controlling genes after transcription [43]. Like miRNAs in Exos, miRNA expression is dependent on external variables and significantly impacts various biological processes. We discovered, through miRNA sequencing, that TE pretreatment might alter the expression of miR-451-5p. Interestingly, a recent study has shown that the miR-451 mimic may reverse the lower cell viability and higher death rate of chondrocytes caused by the overexpression of lncRNA-p21 [44]. More studies are needed to identify the potential regulatory mechanisms for TE pretreatment and miR-451-5p in OA progression.

## 5. Conclusions

We found that Exo^ADSCs^ were able to maintain the chondrocyte phenotype in vitro and promote cartilage repair in OA rats, while TE- Exo^ADSCs^ had comparable or even better performance, possibly due to the upregulation of miR-451-5p induced by the TE pretreatment. In the future, MSC-derived Exos with certain pretreatments or modifications might serve as an effective strategy for treating OA.

## Data Availability

The raw data supporting the conclusions of this article will be made available by the authors, without undue reservation.
